# Genome-wide methylation analysis demonstrates that 5-aza-2-deoxycytidine treatment does not cause random DNA demethylation in fragile X syndrome cells

**DOI:** 10.1186/s13072-016-0060-x

**Published:** 2016-03-24

**Authors:** Elisabetta Tabolacci, Giorgia Mancano, Stella Lanni, Federica Palumbo, Martina Goracci, Fabrizio Ferrè, Manuela Helmer-Citterich, Giovanni Neri

**Affiliations:** Istituto di Medicina Genomica, Università Cattolica del Sacro Cuore, Largo F. Vito 1, 00168 Rome, Italy; Department of Biology, Centre for Molecular Bioinformatics (CBM), University of Rome Tor Vergata, Rome, Italy

**Keywords:** Fragile X syndrome, *FMR1* gene, Epigenetic modifications, DNA methylation, In vitro pharmacological demethylation, Whole methylation analysis, 5-aza-2-deoxycytidine

## Abstract

**Background:**

Fragile X syndrome (FXS) is caused by CGG expansion over 200 repeats at the 5′ UTR of the *FMR1* gene and subsequent DNA methylation of both the expanded sequence and the CpGs of the promoter region. This epigenetic change causes transcriptional silencing of the gene. We have previously demonstrated that 5-aza-2-deoxycytidine (5-azadC) treatment of FXS lymphoblastoid cell lines reactivates the *FMR1* gene, concomitant with CpG sites demethylation, increased acetylation of histones H3 and H4 and methylation of lysine 4 on histone 3.

**Results:**

In order to check the specificity of the 5-azadC-induced DNA demethylation, now we performed bisulphite sequencing of the entire methylation boundary upstream the *FMR1* promoter region, which is preserved in control wild-type cells. We did not observe any modification of the methylation boundary after treatment. Furthermore, methylation analysis by MS-MLPA of *PWS*/*AS* and *BWS/SRS* loci demonstrated that 5-azadC treatment has no demethylating effect on these regions. Genome-wide methylation analysis through Infinium 450K (Illumina) showed no significant enrichment of specific GO terms in differentially methylated regions after 5-azadC treatment. We also observed that reactivation of *FMR1* transcription lasts up to a month after a 7-day treatment and that maximum levels of transcription are reached at 10–15 days after last administration of 5-azadC.

**Conclusions:**

Taken together, these data demonstrate that the demethylating effect of 5-azadC on genomic DNA is not random, but rather restricted to specific regions, if not exclusively to the *FMR1* promoter. Moreover, we showed that 5-azadC has a long-lasting reactivating effect on the mutant *FMR1* gene.

**Electronic supplementary material:**

The online version of this article (doi:10.1186/s13072-016-0060-x) contains supplementary material, which is available to authorized users.

## Background

Fragile X syndrome (FXS; OMIM #300624), the most common cause of inherited intellectual disability, is caused by the absence of FMRP (fragile X mental retardation protein). This loss of function mutation causes dendritic spine dysgenesis [1]. FXS is almost invariably due to a dynamic mutation, i.e. a large expansion (full mutation, FM) of an unstable CGG repeat in the 5′-untranslated region (5′-UTR) of the *FMR1* gene. The CGG expansion is followed by DNA methylation of the 5′-UTR of the gene, which causes *FMR1* transcriptional inactivation and absence of the FMRP protein [2–4]. Despite the knowledge of the epigenetic characteristics of the expanded *FMR1* gene, the molecular mechanisms underlying its silencing are not currently known in detail. The DNA methylation likely represents the main epigenetic mark that switches off the expanded gene. The existence of rare individuals of normal intelligence carriers of unmethylated full mutation (UFM) supports both the crucial role of DNA methylation in silencing the expanded *FMR1* gene and the possibility of transcription of an expanded allele (over 200 CGGs) [5]. Cell lines derived from these individuals might reflect the status of FXS cells before epigenetic silencing, which is thought to occur at about 11 weeks of gestation [6]. Indeed, the epigenetic characterization of their *FMR1* locus showed histone H3 and H4 hyperacetylation, lysine 4 of histone 3 (H3K4) methylation, lysine 9 of histone 3 (H3K9) hypomethylation, lysine 27 of histone 3 (H3K27) dimethylation and lack of DNA methylation [7, 8]. This epigenetic status is compatible with an euchromatic conformation of the *FMR1* locus, allowing transcription. A similar epigenetic status can be induced by treatment of FXS cells with the DNA demethylating agent 5-aza-2-deoxycytidine (5-azadC), which also causes histone changes (H3 and H4 hyperacetylation, H3K4 methylation) that actually precede DNA demethylation [9–11]. In accordance with these results, silencing of *FMR1* in human embryonic stem cells seems to begin from histone modifications prior to DNA methylation [12]. Urbach and colleagues showed that *FMR1* locus in induced pluripotent stem (iPS) cells derived from FXS individuals is hypermethylated, thus suggesting that its methylation, once established, is stable and not revertible through reprogramming techniques [13]. Recently, iPS cells derived from fibroblasts of an UFM individual were found to be methylated after reprogramming, possibly as consequence of in vitro manipulation [14]. The DNA methylation is not widespread, but localized only at the *FMR1* locus in FXS lymphocytes and iPS cells [15]. Naumann et al. demonstrated the presence of a DNA methylation boundary, 650–800 nucleotides upstream of the CGG repeat [16]. This boundary separates, in normal cells, a hypermethylated upstream region from the unmethylated *FMR1* promoter, protecting it from the spreading of DNA methylation and apparently lost in FXS individuals, but not in UFM cell lines [17, 18]. The methylation boundary is thought to have a role in chromatin remodelling of the *FMR1* locus by recruiting a number of proteins [16], such as CTCF (CCCTC-binding factor), the first insulator protein found in mammals [19]. Its role in regulating the *FMR1* gene expression was recently defined, suggesting a complex mechanism via chromatin loop formation [17]. CTCF does not bind to methylated FM alleles, and binding is not restored by pharmacological demethylation with 5-azadC. This result might be explained by failure of drug-induced DNA demethylation to reverse all modifications that occur during gene silencing. As observed on *MLH1* and *p16* gene, 5-azadC treatment did not completely restore normal histone code and post-translational modifications of DNA binding proteins to reestablish long-term expression [20, 21]. We previously observed that transcriptionally reactivated FXS cell lines restored epigenetic changes consistent with an euchromatic status, without fully reaching the euchromatic configuration typical of normal control cell lines [11]. We demonstrated that 5-azadC-induced demethylation is partial and transient. After 4 weeks from 5-azadC withdrawal, the *FMR1* promoter resumed its methylated status [10]. We also showed that inhibitors of histone deacetylases potentiate the effect of 5-azadC, without allowing a substantial reactivation of the gene [22]. These experiments suggest that DNA methylation is dominant over histone modifications in determining the transcriptional inactivation of the mutant *FMR1* gene. This conclusion was confirmed by further experiments, studying the modest reactivating effect of valproic acid (VPA), which acts as inhibitor of histone deacetylases without DNA demethylation [23].

In this report, we describe treatments with 5-azadC of WT and FXS lymphoblastoid cell lines to improve our knowledge of the molecular mechanisms through which this compound induces DNA demethylation and *FMR1* transcriptional reactivation. Specifically, the main aim was to evaluate the possible diffusion of demethylation effect to other loci, different from *FMR1.* Additionally, we analysed the reactivating effect over an extended period of time. Our present findings may have implications for the possible use of 5-azadC as a *FMR1* reactivating drug in vivo.

## Results

### Duration of the *FMR1* reactivating effect of 5-azadC

This study aims also at clarifying the duration of 5-azadC effect by measuring the level of reactivation of *FMR1* at different time points throughout 1 month after discontinuation of the treatment.

**Fig. 1:**
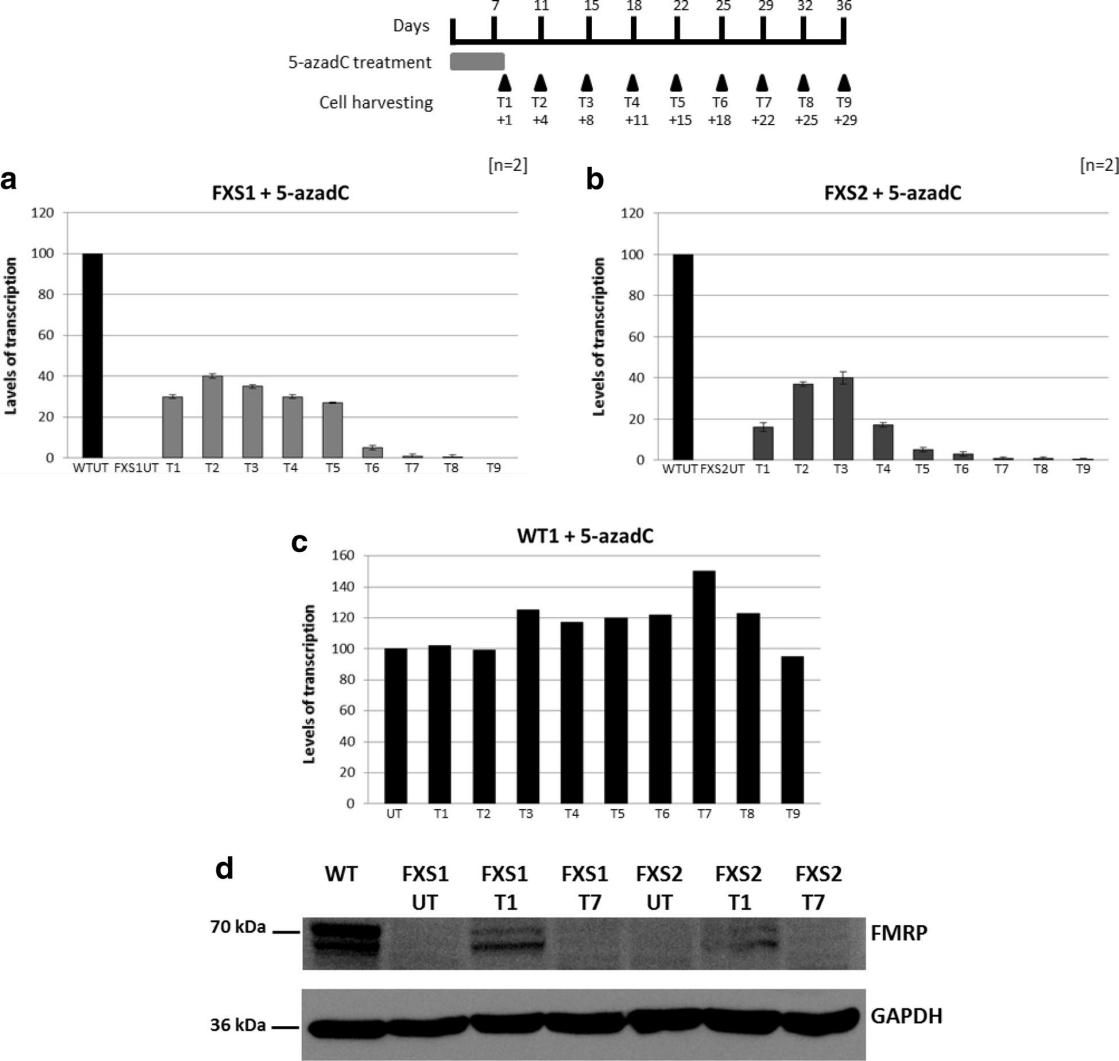
Transcriptional and translational data after 5-azadC treatment. The *upper panel* reports the scheme of 5-azadC treatment with the relative time points. Relative quantification of *FMR1*-mRNA by RT-PCR after two independent treatments (*n* = 2) with 5-azadC of FXS1 (**a**) and FXS2 (**b**) cell lines showed increased *FMR1*-mRNA expression at T2–T3, decreasing at T6 and T7. The reactivation pattern in WT1 cell line was substantially unmodified after 5-azadC treatment (**c**). The transcriptional reactivation levels are expressed in percentage of the untreated WT cell line, arbitrarily set at 100 %. Western blot with antibody against FMRP and GAPDH on FXS1 and FXS2 cell extracts showed that after 7-day treatment with 5-azadC (T1) the expression of FMRP was restored and disappeared after 22 days (T7) from the end of the treatment. After 5-azadC treatment FMRP levels did not reach those of untreated WT

The transcriptional reactivation levels of *FMR1* and the translational expression of FMRP in 5-azadC-treated cell lines are illustrated in Fig. 1. Two independent treatments of FXS1 cell line (around 250 CGGs) induced a reactivation of the *FMR1* transcriptional activity around 30 % with respect to normal control at T1, with a maximum level at T2, T3 and T4 followed by a progressive decrease (Fig. 1a). Two independent treatments on FXS2 cell line (around 450 CGGs) resulted in a reactivation level slightly lower 20 % at T1, with a maximum average reactivation of 40 % at T3, compared with the untreated WT. Afterwards, there was a decrease in transcript, which became undetectable at T7 (Fig. 1b). A slight increase in *FMR1* gene transcriptional activity was observed in 5-azadC-treated WT1 cells, which persisted until 22–25 days after ending the treatment (Fig. 1c). The same trend of transcriptional activity was observed for WT2 cell line (data not shown).

In order to determine whether the newly detected transcriptional activity was paralleled by restoration of the translation, Western blot analysis was performed on protein lysates. We observed a slight restoration of the FMRP expression in FXS1 and FXS2 after 7 days of 5-azadC treatment (T1), while it was no longer detectable after 29 days (T7) (Fig. 1d). This pattern overlaps with that of transcription observed by qRT-PCR. This is the first report of a partial FMRP expression in a FXS cell line after treatment with 5-azadC through Western blot.

**Fig. 2:**
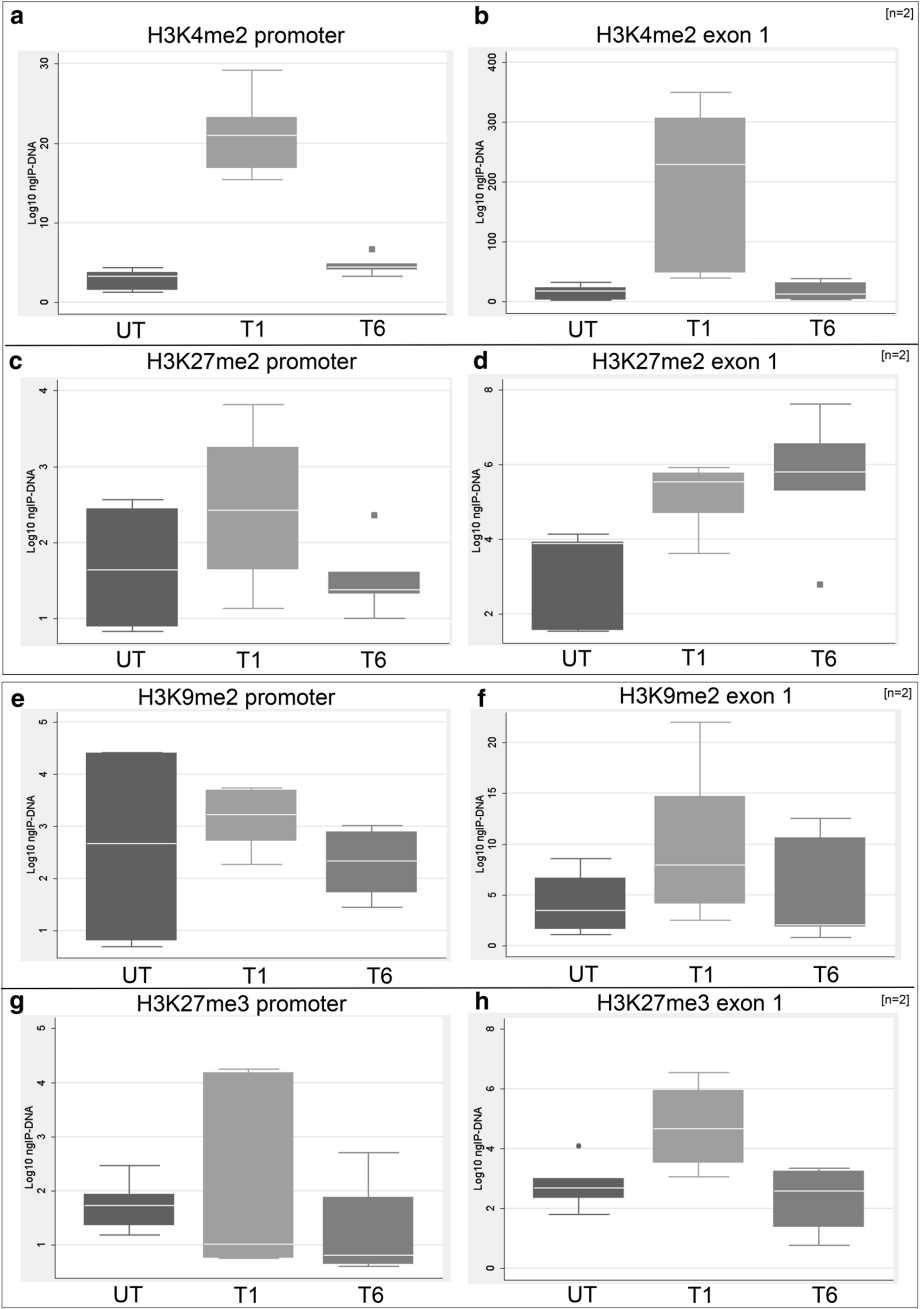
ChIP analysis of euchromatic and heterochromatic markers at the FMR1 locus. Quantification of IP-DNA by real-time PCR after immunoprecipitation with antibodies against euchromatic markers (H3K4me2 and H3K27me2) in the promoter (**a**, **c**) and exon 1 (**b**, **d**) regions of *FMR1*, respectively, positively correlated with the level of transcriptional reactivation observed by quantitative RT-PCR. Heterochromatic markers (H3K9me2 and H3K27me3) in the promoter (**e**, **g**) and exon 1 (**f**, **h**) regions of *FMR1*, respectively, proved to be relatively stable after treatment with 5-azadC even though the trend was in accordance with the transcriptional data. These results refer to two separate ChIP assay performed on cells from two independent experiments on FXS2 cell line at T1 and T6 (*n* = 2). The statistical analysis was conducted using the Kruskal–Wallis nonparametric test (*p* < 0.1)

To study whether the persistent reactivating effect induced by 5-azadC is associated with post-translational histone modifications at the *FMR1* locus, we performed chromatin immunoprecipitation (ChIP) assays followed by quantification of immunoprecipitated DNA (IP-DNA) at different time points. Methylation levels of euchromatic (H3K4me2 and H3K27me2) and heterochromatic (H3K9me2 and H3K27me3) markers of both untreated and treated FXS2 cell line are shown in Fig. 2. The methylation level of H3K4me2 is significantly increased at T1, followed by gradual decrease at T6, both in the promoter and in exon 1 (Fig. 2a, b). The methylation profile of dimethylated H3K27 is similar to that of H3K4me2 at both time points, except for exon 1 region at T6 which persists elevated (Fig. 2c, d). Even if statistically not significant (untreated vs treated, *p* > 0.1), the difference is in the expected direction. Overall, both euchromatic markers show a trend that is concordant with the observed transcriptional activity of the gene. The methylation levels of H3K9me2 were substantially stable (Fig. 2e, f). The observed differences were not statistically significant (*p* > 0.1) at both time points and regions. Despite the lack of statistical significance, the trimethylation level of H3K27 (H3K27me3) in the promoter region was in accordance with the transcriptional activity of *FMR1*, being slightly lower at T1 than at T6 in the promoter region (Fig. 2g). Unexpectedly, we observed a substantial (*p* < 0.1) opposite trend in exon 1 (Fig. 2h).

### Evaluation of the 5-azadC demethylating effect

The study of genomic DNA methylation after 5-azadC treatment was performed by bisulphite sequencing, MS-MLPA of imprinted loci and whole-genome methylation analysis in order to study methylation at different levels, for specific loci and genome wide.

#### Bisulphite sequencing of the methylation boundary

**Fig. 3:**
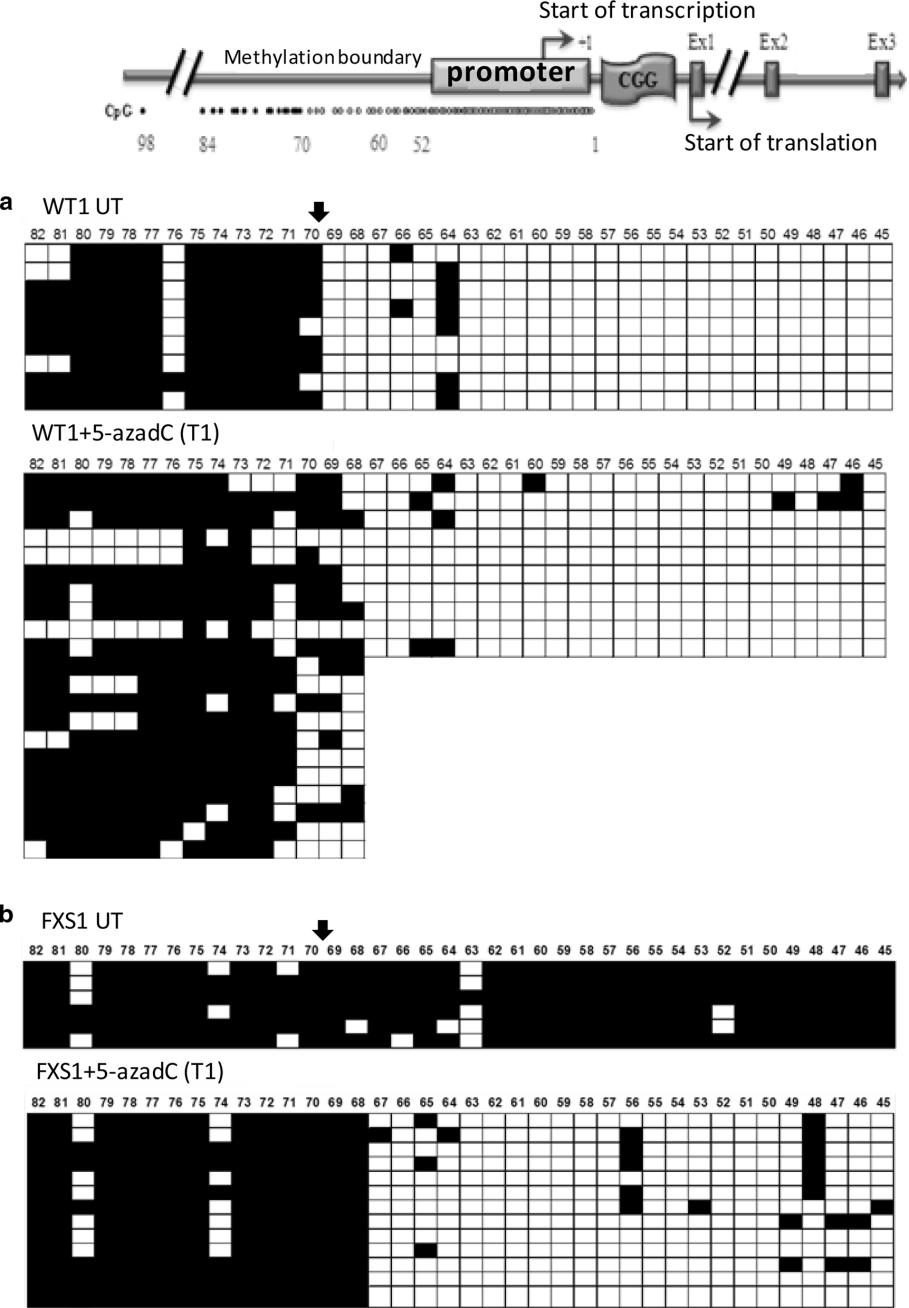
Bisulphite analysis of the FMR1 methylation boundary after 5-azadC treatment. A scheme of the methylation boundary in the 5′ UTR of the *FMR1* locus is reported in the *upper part* of the figure. Bisulphite sequencing of the methylation boundary including the CpG island of the *FMR1* promoter region before (*upper panel*) and after treatment (*bottom panel*) with 5-azadC for 7 days of WT1 revealed no substantial modification of the methylation profile (**a**), while an almost complete demethylation of the promoter region that does not affect the methylation boundary was observed in FXS1 after treatment (**b**). The methylation boundary is indicated by the *arrow*

The demethylating effect of 5-azadC on the genomic regions including part of the CpG island of the *FMR1* promoter (cytosine 45 through 54) and of the methylation boundary [16] was assessed through bisulphite DNA transformation of treated and untreated WT and FXS cell lines. As expected, we observed the presence of the methylation boundary in the untreated WT1 cell line clones, which is lost in the untreated FXS1 cell line (Fig. 3a, b, upper panels), as already described by Naumann et al. [16]. After the treatment with 5-azadC, the methylation profile of WT1 did not substantially change, despite the presence of 3 out of 21 clones unmethylated (Fig. 3a, bottom panel). In the FXS1 cell line, the 5-azadC treatment changed radically the methylation profile of this region: the promoter region became demethylated, as previously demonstrated by Pietrobono et al. [10], while the region upstream the methylation boundary was not affected, preserving its methylated status (Fig. 3b, bottom panel).

FXS2 behaved essentially in the same way (Additional file 1: Figure S1), even though the demethylation effect of the 5-azadC treatment was lower than that observed for the FXS1 cell line, according to the lower levels of *FMR1* transcriptional reactivation and to the CGG expansion of around 450 triplets (Fig. 1b).

#### MS-MLPA of imprinted loci

To further evaluate the methylation status of the *FMR1* locus, as well as of imprinted loci at chromosomes 15 and 11, MS-MLPA was performed in both untreated and 5-azadC-treated cell lines, according to the scheme reported in Fig. 1 (top panel). In particular, MS-MLPA is a semi-quantitative method for methylation profiling, which briefly consists in hybridization of loci-specific probes to the denatured genomic DNA and then digestion with a methylation-sensitive endonuclease.

The first locus analysed was *FMR1* (Additional file 2: Table S1). The methylation profile of the untreated and treated (T1, T3 and T8) WT1 and WT2 cell lines demonstrated the absence of methylation in exon 1, while the other exons retained their methylation status. No substantial changes were observed in the methylation profile after 5-azadC treatment, except for exon 7, which became partially demethylated, at least at T1 and T3. The FXS1 untreated sample had a high methylation level, which was reduced by 5-azadC treatment at T1 and T3, both in exon 1 and exon 7. The methylation level was restored to its normal level at T8. FXS2 cell line behaved in the same way, even though the level of DNA demethylation in exon 1 at T1 was less marked.

In order to have a more comprehensive view of the effect of 5-azadC on other methylated loci, the *PWS/AS* locus on chromosome 15 and the *BWS*/*SRS* locus on chromosome 11 were analysed by MS-MLPA in WT1, WT2, FXS1 and FXS2 cell lines. In all cell lines, we did not observe any substantial change in the methylation profile of either locus after the pharmacological treatment (Tables 1, 2).

**Table 1:** MS-MLPA analysis of the imprinted locus Prader–Willi/Angelman (PWS/AS) on chromosome 15 for WT, WT2, FXS1 and FXS2 before and after treatment with 5-azadC (1 µM) at three different time points (T1, T3 and T8)

Probes	WT1 UT ratio	WT1 T1 ratio	WT1 T3 ratio	WT1 T8 ratio
15-021.5 NDN	***0.48***	***0.30≪***	*0.25≪*	***0.59***
15-022.9 SNRPN	***0.56***	***0.43≪***	***0.51***	***0.48***
15-022.9 SNRPN	***0.57***	***0.35≪***	***0.41***	***0.45***
15-022.9 SNRPN	***0.58***	***0.31*** ≪*	***0.36*** ≪*	***0.5***
15-022.9 SNRPN	***0.41***	*0.18*	***0.3***	*0.22*
15-023.2 UBE3A Exon 1	*0*	*0*	*0*	*0*

Probes	WT2 UT ratio	WT2 T1 ratio	WT2 T3 ratio	WT2 T8 ratio
15-021.5 NDN	***0.48***	*0.24≪*	*0*	***0.37***
15-022.9 SNRPN	***0.52***	***0.4***	***0.47***	***0.61***
15-022.9 SNRPN	***0.52***	***0.39***	***0.44***	***0.55***
15-022.9 SNRPN	***0.5***	***0.36≪***	***0.47***	***0.57***
15-022.9 SNRPN	***0.41***	*0.19≪*	*0.26*	***0.32***
15-023.2 UBE3A exon 1	*0*	*0*	*0*	*0*

Probes	FXS1 UT ratio	FXS1 T1 ratio	FXS1 T3 ratio	FXS1 T8 ratio
15-021.5 NDN	***0.36***	*0.25≪*	*0.28≪*	***0.47***
15-022.9 SNRPN	***0.53***	***0.35***	***0.44***	***0.54***
15-022.9 SNRPN	***0.51***	***0.3***	***0.41***	***0.59***
15-022.9 SNRPN	***0.55***	***0.33<***	***0.39***	***0.53***
15-022.9 SNRPN	***0.4***	*0.14≪*	*0.15≪*	*0.25*
15-023.2 UBE3A exon 1	*0.06*	*0.02*	*0.04*	*0.04*

Probes	FXS2 UT ratio	FXS2 T1 ratio	FXS2 T3 ratio	FXS2 T8 ratio
15-021.5 NDN	***0.48***	***0.31≪***	*0.26≪*	***0.48***
15-022.9 SNRPN	***0.48***	***0.37***	*0.5*	***0.34***
15-022.9 SNRPN	***0.39***	***0.45***	*0.48*	***0.55***
15-022.9 SNRPN	*0.2*	***0.41***	*0.42*	***0.43***
15-022.9 SNRPN	*0.13*	*0.26*	*0.19*	*0.17*
15-023.2 UBE3A exon 1	*0.06*	*0.14≪*	*0*	*0*

Only methylation-sensitive probes are listed

Note that the asterisks indicate that the magnitude of the probe ratio exceed the set of arbitrary border values. The italic values represent levels of methylation lower than 30 %, while bold italic values represent those between 30 and 70 %

**Table 2:** MS-MLPA analysis of the imprinted locus Beckwith–Wiedemann/Silver–Russell (BWS/SRS) on chromosome 11 for WT1, WT2, FXS1 and FXS2 before and after treatment with 5-azadC [1 µM] at three different time points (T1, T3 and T8)

Probes	WT1 UT ratio	WT1 T1 ratio	WT1 T3 ratio	WT1 T8 ratio
11-002.0 H19	*0*	*0*	*0*	*0*
11-002.0 H19	***0.49***	*0.24≪*	*0.26≪*	***0.51***
11-002.0 H19	***0.54***	***0.37≪***	***0.41≪***	***0.57***
11-002.0 H19	***0.49***	*0.28≪*	***0.32≪***	***0.55***
11-002.1 IGF2	*0*	*0*	*0*	*0*
11-002.7 KCNQOT1	***0.53***	***0.38***	***0.39***	***0.57***
11-002.7 KCNQOT1	***0.46***	*0.28≪*	***0.33≪***	***0.51***
11-002.7 KCNQOT1	***0.45***	***0.33***	***0.37***	***0.54***
11-002.7 KCNQOT1	***0.41***	***0.3***	***0.37***	***0.58***
11-002.9 CDKN1c	*0.17*	*0.18*	*0.2*	*0.15*

Probes	WT2 UT ratio	WT2 T1 ratio	WT2 T3 ratio	WT2 T8 ratio
11-002.0 H19	*0.23*	*0*	*0*	*0*
11-002.0 H19	***0.55***	*0.26≪*	*0.26≪*	***0.47***
11-002.0 H19	***0.54***	***0.37≪***	***0.35≪***	***0.5***
11-002.0 H19	***0.52***	***0.35***	***0.36***	***0.51***
11-002.1 IGF2	*0*	*0*	*0*	*0*
11-002.7 KCNQOT1	***0.48***	***0.41***	*0.29≪*	***0.59***
11-002.7 KCNQOT1	***0.44***	***0.33≪***	*0.22≪*	***0.52***
11-002.7 KCNQOT1	***0.42***	***0.34***	*0.26≪*	***0.5***
11-002.7 KCNQOT1	***0.42***	***0.35***	*0.18≪*	***0.56***
11-002.9 CDKN1c	*0.13*	*0.15*	*0*	*0.15*

Probes	FXS1 UT ratio	FXS1 T1 ratio	FXS1 T3 ratio	FXS1 T8 ratio
11-002.0 H19	*0.23*	*0.05*	*0.07*	*0.07*
11-002.0 H19	***0.54***	*0.1≪*	*0.09≪*	*0.1*
11-002.0 H19	***0.62***	***0.42≪***	***0.47<***	***0.59***
11-002.0 H19	***0.59***	***0.32≪***	***0.45***	***0.49***
11-002.1 IGF2	*0.02*	*0.03*	*0.02*	*0.02*
11-002.7 KCNQOT1	***0.59***	***0.42***	***0.54***	***0.6***
11-002.7 KCNQOT1	***0.59***	***0.33≪***	***0.44***	***0.54***
11-002.7 KCNQOT1	***0.51***	***0.42***	***0.53***	***0.5***
11-002.7 KCNQOT1	***0.54***	***0.36***	***0.49***	***0.58***
11-002.9 CDKN1c	*0.1*	*0.13*	*0.16*	*0.1*

Probes	FXS2 UT ratio	FXS2 T1 ratio	FXS2 T3 ratio	FXS2 T8 ratio
11-002.0 H19	*0*	*0*	*0.19*	*0*
11-002.0 H19	*0.26*	*0.22*	*0.23*	*0.29*
11-002.0 H19	***0.67***	***0.37≪***	***0.42≪***	***0.47***
11-002.0 H19	0.71	***0.47≪***	***0.34≪***	0.77
11-002.1 IGF2	*0.19*	*0.14*	*0.11*	*0.18*
11-002.7 KCNQOT1	***0.5***	***0.34***	***0.68***	***0.6***
11-002.7 KCNQOT1	***0.4***	***0.42***	***0.44***	***0.42***
11-002.7 KCNQOT1	***0.55***	***0.4***	0.74	***0.58***
11-002.7 KCNQOT1	***0.5***	***0.47***	***0.44***	0.92
11-002.9 CDKN1c	*0.25*	*0.28*	***0.3***	***0.35***

Only methylation-sensitive probes are listed

The italic values represent levels of methylation lower than 30 %, bold italic values represent those between 30 and 70 %, while underlined values represent those higher than 70 %

Additionally, to rule out a possible effect of the demethylating agent on the inactive (methylated) X chromosome, we also treated three different female WT cell lines (WTA, WTB and WTC) with 5-azadC and subsequently analysed the methylation profile of *FMR1* by MS-MLPA (Table 3). The results of this analysis of the two imprinted loci *PWS/AS* and *BWS/SRS* are detailed in Additional file 3: Table S2 and Additional file 4: Table S3. Once again, the pharmacological treatment does not seem to affect the methylation levels in any of the analysed regions.

**Table 3:** MS-MLPA analysis on the *FMR1* locus before and after 7-day treatment with 5-azadC (T1) of three different normal control female cell lines (WTA, B and C)

Probes	WTA UT ratio	WTA T1 ratio	WT B UT ratio	WT B T1 ratio	WTC UT ratio	WTC T1 ratio
Prom	***0.48***	***0.38***	***0.52***	***0.3≪***	***0.52***	***0.37≪***
Prom	***0.47***	*0.25*	***0.42***	***0.32***	***0.53***	*0.25*
Exon 1	***0.33***	*0.26*	*0.27*	***0.3***	***0.39***	*0.28*
Intron 1	***0.56***	***0.36***	***0.52***	*0.19≪*	***0.68***	***0.42***
Intron 1	***0.25***	*0.19*	***0.3***	***0.4***	***0.38***	*0.23*
Intron 1	***0.36***	*0.24*	***0.35***	*0.25*	***0.35***	*0.25*
Intron 1	***0.35***	*0.24*	***0.3***	*0.25*	***0.39***	*0.26*
Exon 7	0.98	***0.46*** ≪*	0.98	***0.6*** ≪*	0.93	***0.58*** ≪*
Exon 17	0.97	0.9	0.94	0.93	0.97	0.88
Exon 1 AFF2	***0.52***	***0.45***	***0.54***	***0.43≪***	***0.61***	***0.42≪***
Exon 1 AFF2	***0.49***	***0.37***	***0.42***	***0.33***	***0.42***	***0.33***
Exon 1 AFF2	*0.23*	*0.23*	***0.3***	*0.19≪*	***0.34***	*0.24*
Exon 1 AFF2	***0.46***	***0.3***	***0.49***	***0.36***	***0.53***	***0.38***
Exon 1 AFF2	*0.27*	*0.17*	*0.22*	*0.14*	***0.38***	*0.23*
Xp22	***0.41***	***0.34***	***0.38***	*0*	*0*	*0*

Only methylation-sensitive probes are listed

Note that the asterisks indicate that the magnitude of the probe ratio exceeds the set of arbitrary border values. The italic values represent levels of methylation lower than 30 %, bold italic values represent those between 30 and 70 %, while underlined values represent those higher than 70 %

#### Whole-genome methylation analysis

In order to investigate the demethylating effect of 5-azadC on global DNA, we performed a whole methylation study (methylome) using Infinium Human Methylation 450 BeadChip array. The methylation degree of each CpG sites is defined by the *β* value and ranges between 0 (not methylated) and 1 (methylated). Each analysed sample shows two major peaks of CpG methylation value, i.e. for *β* = 0 and 0.8 < *β* < 1 (Additional file 5: Figure S2).

In order to exclude an ascertainment bias due to possible differences in the baseline methylation levels of lymphoblastoid cell lines, we compared our data with data published by Alisch et al. [15], who examined genomic DNA methylation levels of peripheral blood and fibroblast-derived iPS cells of FXS patients using the Infinium Human Methylation 450 BeadChip array. The statistical analysis was performed using the Pearson correlation coefficient, which gives a value between +1 and −1 inclusive, where 1 is total positive correlation, 0 is no correlation and −1 is total negative correlation. In our case, the coefficient ranged from 0.6 to 0.85, showing a high positive correlation between the methylation levels of our cell lines (numbered from 1 to 9, as detailed in the “Methods” section) and the peripheral blood samples of Alisch et al. [15] (Additional file 6: Figure S3).

**Fig. 4:**
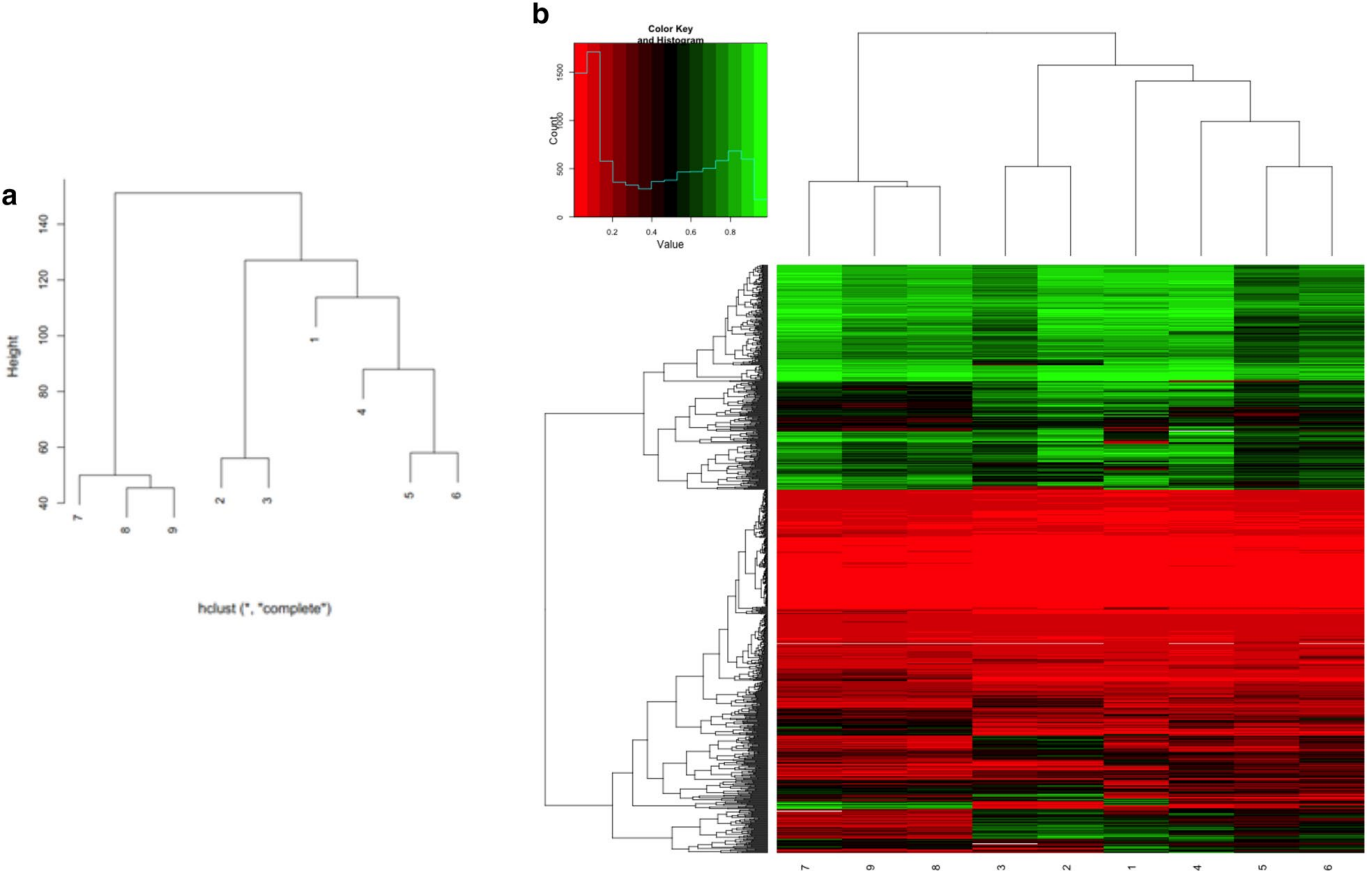
Dendrogram and heat map of the methylation profile in the analysed samples. Dendrogram of the nine samples demonstrates a clustering of samples 4–6 (FXS1 UT and the two different 5-azadC treatments, respectively) and 7–9 (FXS2 UT and the two different 5-azadC treatments, respectively), as expected (**a**). The distance between sample 1 (WT2 UT) and samples 2 and 3 (WT1 UT and WT1 T1, respectively) and the similarity between samples 2 and 3 can be explained by intrinsic difference and similarities between different cell lines. The closeness between sample 1 (WT2 UT) and 4 (FXS1 UT) confirms the observations by Alisch et al. [15]. Heat map of the methylation profile of randomly sampled *loci* of all analysed samples shows that samples from the same cell line type clustered together (**b**). It is possible to observe some changes in the methylation profile after treatment with 5-azadC that do not reach statistical significance (*p* > 0.05)

The clustering analysis of the nine samples shows the distance and similarity among different cell lines and within different samples of each cell line compared with the respective untreated one (Fig. 4a). As shown in Fig. 4b, after treatment with 5-azadC it is possible to observe changes in the methylation profile of each treated sample. However, these modifications are not statistically significant (*p* > 0.05).

**Fig. 5:**
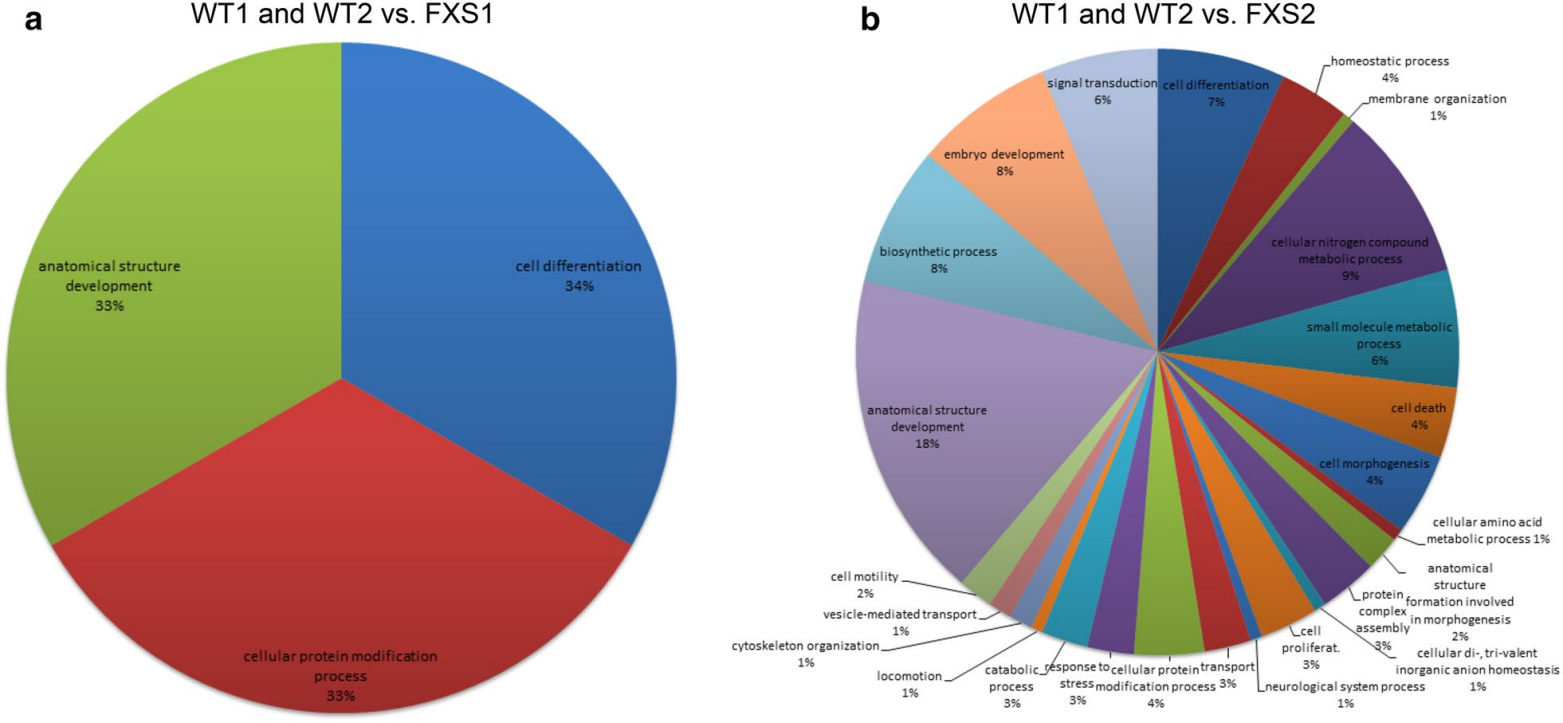
Comparison between samples with respect to GO terms. In **a** is reported the comparison between WT1 and WT2 versus untreated FXS1. Note the enrichment of DMRs for only three GO terms. There is a broad distribution of DMRs over several GO terms in the comparison between WT1 and WT2 versus FXS2, although it is possible to identify two major enriched ones (anatomical structure development and cellular nitrogen compound metabolic process) (**b**)

Significantly enriched Gene Ontology (GO) terms were summarized through the GO slim subsets in Fig. 5, and a schematic list of differentially methylated regions (DMRs) with the number of genes content is reported in Table 4. The comparison of the DMRs between untreated WT (WT1 and WT2) and FXS1 demonstrated an enrichment for a few specific GO terms (Fig. 5a). The comparison between untreated WT (WT1 and WT2) and FXS2 (Fig. 5b) revealed a discrete enrichment of a few specific GO terms. Of note, the analysis of FXS1 and FXS2 cell lines showed no DMRs in the comparison between the two corresponding treated samples. The complete list of DMRs in the two comparisons illustrated in Fig. 5 with their chromosome position is reported in Additional file 7: Table S4 and Additional file 8: Table S5.

**Table 4:** List of differentially methylated sites (DMS), differentially methylated regions (DMRs) and genes content in the four comparisons

Comparison	DMS	DMR	Genes
WT1 and WT2 versus FXS1	7343	40	32
WT1 and WT2 versus FXS2	33,950	365	300
FXS1 UT versus FXS1 T1 (*n* = 2)	1706	0	0
FXS2 UT versus FXS2 T1 (*n* = 2)	528	0	0

## Discussion

Many efforts have been made in terms of possible pharmacological approaches to FXS, aiming either at mimicking FMRP functions or at restoring the transcriptionally active epigenetic profile of *FMR1* [24].

*In vitro* studies directed at restoring *FMR1* activity by reinstating its active epigenetic profile showed promising results [9–11, 23, 25], leading to a couple of pilot clinical trials [26, 27].

The major objection to use drugs like 5-azadC or HDAC inhibitors is that their action could be unspecific and genome wide, with unforeseeable damaging consequences, even though a microarray screening of 10,814 genes showed that a very limited set of genes are actually transcriptionally upregulated by treatment with 5-azadC (51 genes) and/or with trichostatin A (23 genes) [28].

To shed more light on this debated question, we extended our previous studies on the epigenetics of the *FMR1* promoter to other genomic regions and eventually to the entire genome. Although only H3K4me2 data reached statistical significance (*p* < 0.01), the overall picture of ChIP assay results showed a consistency of the euchromatic markers (H3K4me2 and H3K27me2) with the transcriptional data, while the heterochromatic markers (H3K9me and H3K27me3) did not change substantially during the transition of the *FMR1* gene from transcriptionally inactive to transcriptionally active. These last data confirm and extend previous findings, which showed that the methylation level of H3K9me2 is not substantially affected by treatment with 5-azadC and that, in UFM cell lines, the epigenetic profile of this marker was more similar to that of the FXS cells [11, 23].

To complete the evaluation of the epigenetic effects of 5-azadC, as well as concerns about its lack of specificity, we used three different approaches to investigating the changes in DNA methylation at different levels: (1) site specific (bisulphite sequencing); (2) locus specific (MS-MLPA); (3) global (whole methylation analysis).

The bisulphite sequencing analysis of the methylation boundary [16] and of the promoter region of *FMR1* disclosed that while the promoter region of FXS cells underwent a substantial demethylation, as already observed [10], the methylation boundary was not affected by treatment with 5-azadC. This new finding suggests that 5-azadC does not act indiscriminately throughout genomic DNA and that constitutively methylated regions are somehow “protected” from its demethylating effect. To confirm the validity of this concept, we checked the methylation status of other genomic sites, specifically the two well-known imprinted loci *PWS/AS* and *BWS/SRS*, through MS-MLPA. The methylation profile of both loci remained substantially unchanged after 5-azadC treatment. MS-MLPA analysis of the *FMR1* locus and of the two imprinted loci analysed in three different WT female cell lines did not show any significant modification after the demethylating treatment. On the whole, these data strengthen the assumption that some genomic regions are indeed shielded from the demethylating action of 5-azadC. Finally, the whole methylation analysis showed that the methylation changes induced by 5-azadC did not affect significantly DMRs. A broad distribution of several GO terms or a discrete distribution of few of them was observed (Fig. 5; Table 4). More importantly, we observed a very strong positive correlation (*p* < 0.000001) between the methylation profiles of lymphoblastoid cell lines employed in this study and that of a set of different peripheral blood samples (and iPS cell lines) described by Alisch et al. [15]. These data argue against the possibility that the lack of methylation changes after treatment with 5-azadC may be interpreted as due to a different pattern of DNA methylation in lymphoblastoid cell lines, compared with peripheral blood cells. In fact, different comparative genome-wide DNA methylation studies between lymphocytes and their derived lymphoblasts demonstrated differences in methylation profile of both cell line types. The direction of these changes in DNA methylation was often not consistent, i.e. hypermethylation of some regions and hypomethylation of others in lymphoblasts with respect to lymphocytes. These DMRs seemed to reflect differences in methylation between B lymphocytes and other subtypes of white blood cells, considering the monoclonal nature of lymphoblasts and their modifications throughout virus transformation and culturing procedure [29–31]. Nevertheless, another study showed a high correlation between lymphocytes and virus-transformed lymphoblasts from the same donor in terms of DNA methylation and transcription pattern [32]. In any case, the strong correlation we observed between lymphoblastoid cell lines and peripheral blood cells supports the consistency and validity of our results.

To date, 5-azadC is the only compound known to induce a good transcriptional activity of the expanded and silenced *FMR1* gene. Previous experiments with 5-azadC investigated the transcriptional activity of *FMR1* after 7 days of administration, when the levels of *FMR1* mRNA increased to 30–40 % of a WT cell line, and again at 30 days after the last dose administration, when complete absence of transcriptional activity, due to the remethylation of the CpG island of the promoter region, was observed [10]. Now we have investigated the changes in *FMR1* transcription level occurring between day 1 and day 30 after cessation of the treatment, in order to measure its effective duration. Both FXS cell lines showed the expected transcriptional reactivation that, interestingly, persisted for about 10–15 days after the last dose of 5-azadC. The WT cell line did not show any significant changes in *FMR1* transcriptional level after treatment. These results suggest that the effect of 5-azadC on *FMR1* transcription is only temporary, but relatively long-lasting, requiring only pulsed administrations, in order to keep reactivation of *FMR1* in a steady state. One clear implication of this finding is the containment of possible damaging side effects of 5-azadC.

The administration of 5-azadC to FXS patients has not been tried so far. An obvious concern that arises when considering the clinical use of 5-azadC is its toxicity. In fact, while 5-azacytidine (5-azaC) and 5-azadC are generally well tolerated in haematological malignancies [33], the effects of a long-term treatment on patients affected by genetic disorders are unknown. The results presented here suggest that use of 5-azadC in a hypothetical trial to treat FXS may not require daily administrations of the drug, thus limiting its possible undesirable side effects. The idea of treating a genetic disease with a demethylating drug is not new. Besides haematological malignancies, 5-azadC has been used to treat patients with severe refractory β-thalassemias, with a reexpression of foetal haemoglobin [34]. Moreover, few in vitro treatments with 5-azadC of genetic syndromes have been reported. Yao et al. [35] have treated with 5-azadC three primary human BWS cell lines, two *β2SP* negative and one *β2SP* positive. The non-pleckstrin homology domain β-spectrin (*β2SP*) functions as a potent TGF-β signalling member adaptor and is epigenetically silenced by DNA methylation of its CpG island in many individuals with BWS without loss of imprinting at the *IGF2* locus. Treatment with 5-azadC caused the reactivation of the *β2SP* expression in two negative BWS cell lines correlating with a decrease in DNA methylation in both *β2SP* alleles [35].

A second obstacle to the therapeutic approach with 5-azadC is the apparent requirement of cell division in order for the treatment to be effective. Interestingly, at least two reports suggest that 5-azadC may require minimal or no incorporation into DNA to effectively reduce the activity of the maintenance DNA methyltransferase DNMT1 [36, 37]. Furthermore, Bar-Nur et al. [38] treated FXS-iPS cells and their derived neurons with 5-azaC and observed a robust *FMR1* reactivation after treatment. Although it was a pilot study, these authors demonstrated that an epigenetic intervention in stable nervous cells is possible.

## Conclusions

We show for the first time that 5-azadC seems to have a discreet level of specificity, with a long-lasting effect. In our view, these results call for future studies on FXS-iPS cells and their derived neurons, which we consider an obligate pre-clinical passage before 5-azadC can be tested in a clinical trial.

## Methods

### Cell lines and treatments

Lymphoblastoid cell lines were established through Epstein–Barr virus (EBV) immortalization of peripheral blood lymphocytes of two FXS individuals, two normal control males (WT) and three normal control females. Peripheral blood lymphocytes were obtained after a signed informed consent of each participant (or parents in the case of FXS patients). Ethics Committee at the Catholic University of Rome approved this study (prot. N. 9917/15 and prot.cm 10/15). All cell lines had been anonymously established in the Institute of Genomic Medicine at the Catholic University (Rome) and are referred to as follows:

- FXS1, fragile X syndrome cell line 1, around 250 CGG repeats;
- FXS2, fragile X syndrome cell line 2, around 450 CGG repeats;
- WT1, wild-type cell line 1, normal control male;
- WT2, wild-type cell line 2, normal control male;
- WT A, wild-type cell line A, normal control female;
- WT B, wild-type cell line B, normal control female;
- WT C, wild-type cell line C, normal control female.

Cell cultures were grown in RPMI1640 medium (Sigma-Aldrich), supplemented with 10 % foetal bovine serum (FBS), 2.5 % penicillin/streptomycin and 2.5 % l-glutamine at 37 °C with 5 % CO_2_.

Prior to pharmacological treatment, cell viability was assessed through propidium iodide staining (NucleoCounter, Sartorius-Stedim). For each cell culture, we seeded 9 × 10^7^ cells/ml in a final volume of 130 ml per single flask. 5-AzadC (Sigma-Aldrich, A3656) was added to the cell cultures (final concentration 1 µM) for 7 consecutive days. The day after the last dose administration, an aliquot of the cell suspension was harvested for RNA, DNA and protein extraction and for chromatin immunoprecipitation (ChIP) assays. In order to evaluate the long-lasting effect of the treatment, the remaining volume was subdivided into eight different flasks, and the cultures continued according to the scheme reported on the top panel of Fig. 1. For each WT cell line, one single treatment was performed, while two independent treatments were performed for each FXS cell line (*n* = 2).

### Quantitative RT-PCR

Total RNA was extracted by RNeasy mini kit (Qiagen, 74106). RNA concentration and purity were checked on 1.5 % agarose gel and by UV spectrophotometer. Afterwards, 500 ng of total RNA was retro-transcribed into cDNA by MoMLV-RT (Invitrogen, USA) and 0.6 µg of random hexamers (Promega, C118A).

For a relative quantification of each transcript using ABI7900HT (Life technologies), the following pre-developed TaqMan assays were used: *FMR1* (Hs00233632_m1) and *GAPDH* (glyceraldehyde-3-phosphate dehydrogenase) (Hs02758991_g1), the latter being constitutively expressed in every cell and thus used as endogenous control. The cycle parameters were: 2 min at 50 °C and 10 min at 95 °C, followed by 40 cycles with 15 s at 95 °C (denaturation) and 1 min at 60 °C (annealing/extension). The relative quantification of *FMR1* transcript versus *GAPDH* transcript was calculated as follows: 2^−[ΔCt(*FMR1*)−ΔCt(*GAPDH*)]^ = 2^−ΔΔCt^, being ΔCt the difference [Ct(*FMR1*) − Ct(*GAPDH*)] and Ct the cycle at which the detected fluorescence overcomes the threshold.

### Western blot

Proteins extracted from untreated and 5-azadC-treated FXS1 and FXS2 and WT1 lymphoblasts were resuspended in Laemmli buffer, boiled, separated on 8 % polyacrylamide gel electrophoresis, transferred to Hybond-ECL membrane (GE Healthcare), immunostained and visualized after film exposure using the ECL Western blotting kit (GE Healthcare), according to the manufacturer. Primary antibodies were used at the following concentrations: 1:1000 anti-FMRP mouse polyclonal antibody (Immunological Science) and 1:10,000 anti-GAPDH mouse antibody (Sigma-Aldrich).

### Chromatin immunoprecipitation assay and quantification of IP-DNA

To study the histone modifications at the *FMR1* locus, chromatin immunoprecipitation (ChIP) assay was performed according to the manufacturer (Millipore). Histone methylation analysis was performed using two different antibodies against dimethyl lysine 9 (H3K9me2, Millipore), dimethyl lysine 4 (H3K4me2, Millipore) and di- and trimethyl lysine 27 (H3K27me2 and H3K27me3, Millipore) on histone 3. In each ChIP assay, antibody against rabbit IgG (Thermo Scientific, 1862244) was employed and no template control was included. Immunoprecipitated DNA (IP-DNA) was extracted by standard procedure (phenol/chloroform/isoamilic alcohol 25:24:1) and then quantified by real-time PCR (ABI7900HT, Life Technologies) using fluorescent probes and primers specific for both *FMR1* and *GAPDH*, as already published [8].

Standard curves for the two *FMR1* and for the single *GAPDH* amplicons were constructed with five different DNA dilutions of known concentration [*X* axis = log(*X*)] and the corresponding Ct values (*Y* axis). The unknown amount of IP-DNA of *FMR1* and *GAPDH* [*X* axis = log(*X*)] was calculated from Ct values, through the standard curve plot [*y* = *ax* + *b*, that is Ct = *ax* + *b*, with *x* = log(*X*)].

All variables were analysed by means of descriptive statistics (mean, median, standard deviation and standard error of mean). Data were analysed with nonparametric statistical Kruskal–Wallis test and with K sample test. The level of significance was set at *p* ≤ 0.01. Data analysis was performed using STATA Intercooled version 9.2 software (Stata Co.; College Station, Lakeway, TX, USA).

### Bisulphite sequencing

Genomic DNA was isolated from treated and untreated cells by DNeasy Blood & Tissue kit (Qiagen, 69504). The DNA concentration was checked both by absorbance measurements at 260 nm and on agarose gel. Bisulphite DNA transformation was performed as previously described [17]. Each transformed DNA was amplified in seven independent PCR reactions, then pooled and recovered from the agarose gel with the StrataPrep DNA Gel extraction kit (Stratagene, 400768). The purified PCR products were cloned with the StrataClone PCR cloning kit (Stratagene, 240205), according to the manufacturer’s instructions. After bacterial plating and overnight incubation at 37 °C, white colonies were picked and plasmid DNA was extracted. After a pre-screening of the clones with PCR using specific plasmid primers (T3 and T7), amplification products were sequenced in both directions with BigDye Terminator version 3.1 Cycle Sequencing kit (Life Technologies, 4336917) on a 3130 Genetic Analyzer (Life Technologies). The modified primers are those described by Naumann et al. [16].

### MS-MLPA analysis

Evaluation of DNA methylation through MS-MLPA was performed according to the manufacturer protocol (MRC Holland). In the MS-MLPA assay, the sequence targeted by specific probes contains a restriction site for the *Hha*I endonuclease, able to recognize the unmethylated GCGC sequence. Fragment separation was performed through capillary electrophoresis (ABI3130 Genetic Analyzer, Life Technologies), and the methylation status was established through Coffalyser DB software, comparing the signal of each probe (that is proportional to the amount of target DNA) in each sample after *Hha*I digestion with the signal of the same probe without digestion. At least three reference samples (untreated WT) were used in all experiments to normalize the data obtained for each test probe of each sample. Based on the methylation status of these reference samples, the Coffalyser DB software automatically determines the border allowing for statistical analysis. Reference samples were randomly distributed across the plate, to calculate the reproducibility of each probe in the reference sample population. No DNA controls were included. The genomic regions analysed in the current study are: *FMR1*-*AFF2* locus in Xq27.3 (ME029B2), Prader–Willi/Angelman syndromes (*PWS/AS*) locus in 15q11 (ME028B2) and Beckwith–Wiedemann/Silver–Russell syndromes (*BWS/SRS*) locus in 11p15 (ME030C1).

### Whole-genome methylation analysis

Whole methylation study was performed through Infinium Human Methylation 450 BeadChip array in out-service (Integragen). This array detects cytosine methylation at CpG islands based on highly multiplexed genotyping of bisulphite-converted genomic DNA. The level of methylation for the interrogated locus can be determined by calculating the ratio of the fluorescent signals from the methylated versus unmethylated sites. The raw data are analysed by the proprietary software, and the fluorescence intensity ratios between two bead types are calculated. A ratio value of 0 equals to non-methylation of the locus, a ratio of 1 equals to total methylation, a value of 0.5 means that one copy is methylated, and the other is not, in the diploid human genome (Human Methylation 450 BeadChip Data Sheet; Illumina website). For the present study, nine samples were analysed and numbered from 1 to 9, respectively, as follows: untreated WT2, untreated WT1 and 7-day treatment of WT1 (WT1 T1), untreated FXS1 and two independent 7-day treatments (FXS1 T1) (*n* = 2), untreated FXS2 and two independent 7-day treatments (FXS2 T1) (*n* = 2). Differential methylation was analysed using the minfi package [39]. Data were background-corrected and then subject to subset-quantile within array normalization (SWAN) [40]. Differentially methylated positions were identified using the minfi *F* test implementation and selected as those having *p* value < 0.01. Differentially methylated regions were identified using the lasso algorithm [41] implemented in the ChAMP package [42], by submitting to ChAMP the minfi *F* test output. A threshold of 0.05 was set for the adjusted *p* value. Genes associated with methylation sites found in significantly differentially methylated regions, as retrieved from the Illumina documentation, were annotated for functional enrichment by accessing the David web services [43] through the openpyxl Python library (https://openpyxl.readthedocs.org/en/latest/). Significantly enriched Gene Ontology terms were summarized through the Gene Ontology (GO) slim subsets.

In order to compare our data set from lymphoblastoid cell lines with those already published by Alisch et al. [15] from lymphocytes and iPS cell lines, we used the Pearson correlation coefficient.

## Additional files

10.1186/s13072-016-0060-x Bisulphite sequencing of the FMR1 methylation boundary after 5-azadC treatment in FXS2 cell line. Partial demethylation after treatment with 5-azadC is limited to the CpG island of the promoter region (middle panel), even after 8 days (T1) from 5-azadC withdrawal (bottom panel). The arrow indicates the position of the methylation boundary.
10.1186/s13072-016-0060-x MS-MLPA analysis of the FMR1/AFF2 locus before and after 5-azadC treatment at three different time points (T1, T3 and T8) of two different normal control cell lines (WT1 and WT2), two different treatments of FXS1 and FXS2, respectively. Only methylation-sensitive probes are listed.
10.1186/s13072-016-0060-x MS-MLPA analysis of the PWS/AS locus on chromosome 15 before and after 7-day treatment with 5-azadC (T1) of three different normal control female cell lines (WTA, B and C). Only methylation-sensitive probes are listed.
10.1186/s13072-016-0060-x MS-MLPA analysis of the BWS/SRS locus on chromosome 11 before and after 7-day treatment with 5-azadC (T1) of three different normal control female cell lines (WTA, B and C). Only methylation-sensitive probes are listed.
10.1186/s13072-016-0060-x Distribution curve of beta values for each of nine different samples. Two main peaks, for beta = 0 and 0.8 < beta < 1, are visible; beta = 0 means absence of DNA methylation and beta = 1 means completely methylated DNA.
10.1186/s13072-016-0060-x Heat map of the Pearson correlation analysis. Heat map illustrating the Pearson correlation coefficient between the nine different lymphoblastoid cell lines used for this study (columns from 1 to 9) and the data set of peripheral blood and iPS cells published by Alisch et al. [2013] (rows). A high correlation has been demonstrated with values ranged from 0.6 and 0.85 between our lymphoblastoid cell lines and those already published. Statistical significance was reached (*p* < 0.000001).
10.1186/s13072-016-0060-x List of DMRs and their chromosome position in the comparison between WT1 and WT2 versus FXS1.
10.1186/s13072-016-0060-x List of DMRs and their chromosome position in the comparison between WT1 and WT2 versus FXS2.

## Abbreviations

5-azadC: 5-aza-2-deoxycytidine
PWS/AS: Prader–Willi/Angelman syndrome
BWS/SRS: Beckwith–Wiedemann/Silver–Russell syndrome
VPA: valproic acid
