# Supplementary material for: Genome-wide methylation analysis demonstrates that 5-aza-2-deoxycytidine treatment does not cause random DNA demethylation in fragile X syndrome cells
Source: Epigenetics Chromatin. 2016 Mar 24;9:12. doi: 10.1186/s13072-016-0060-x (PMC4806452; doi:10.1186/s13072-016-0060-x)
Supplement: Supplementary file 1 — 10.1186/s13072-016-0060-x Bisulphite sequencing of the FMR1 methylation boundary after 5-azadC treatment in FXS2 cell line. Partial demethylation after treatment with 5-azadC is limited to the CpG island of the promoter region (middle panel), even after 8 days (T1) from 5-azadC withdrawal (bottom panel). The arrow indicates the position of the methylation boundary. [file 13072_2016_60_MOESM1_ESM.docx]

**Additional file: Figure S1.** *Bisulphite sequencing of the FMR1 methylation boundary after 5-azadC treatment in FXS2 cell line.* Partial demethylation after treatment with 5-azadC is limited to the CpG island of the promoter region (middle panel), even after 8 days (T1) from 5-azadC withdrawal (bottom panel). The arrow indicate the position of the methylation boundary.
