# Supplementary material for: Genome-wide methylation analysis demonstrates that 5-aza-2-deoxycytidine treatment does not cause random DNA demethylation in fragile X syndrome cells
Source: Epigenetics Chromatin. 2016 Mar 24;9:12. doi: 10.1186/s13072-016-0060-x (PMC4806452; doi:10.1186/s13072-016-0060-x)
Supplement: Supplementary file 2 — 10.1186/s13072-016-0060-x MS-MLPA analysis of the FMR1/AFF2 locus before and after 5-azadC treatment at three different time points (T1, T3 and T8) of two different normal control cell lines (WT1 and WT2), two different treatments of FXS1 and FXS2, respectively. Only methylation-sensitive probes are listed. [file 13072_2016_60_MOESM2_ESM.docx]

**Additional file: Table S1.** MS-MLPA analysis of the *FMR1/AFF2* locus before and after 5-azadC treatment at three different time points (T1, T3 and T8) of two different normal control cell lines (WT1 and WT2), two different treatments of FXS1 and FXS2, respectively. Only methylation-sensitive probes are listed.

| **Probes** | **WT1 UT** | **WT1 T1** | **WT1 T3** | **WT1 T8** | **WT2 UT** | **WT2 T1** | **WT2 T3** | **WT2 T8** |
| --- | --- | --- | --- | --- | --- | --- | --- | --- |
|  | ratio | ratio | ratio | ratio | ratio | ratio | ratio | ratio |
| Prom | 0 | 0 | 0 | 0 | 0 | 0 | 0 | 0 |
| Prom | 0 | 0 | 0 | 0 | 0 | 0 | 0 | 0 |
| Exon 1 | 0 | 0 | 0,18>> | 0 | 0 | 0 | 0,18>> | 0 |
| Intron 1 | 0 | 0 | 0 | 0 | 0 | 0 | 0 | 0 |
| Intron 1 | 0 | 0 | 0 | 0 | 0 | 0 | 0 | 0 |
| Intron 1 | 0 | 0 | 0 | 0 | 0 | 0 | 0 | 0 |
| Intron 1 | 0 | 0 | 0 | 0 | 0 | 0 | 0 | 0 |
| Exon 7 | 1,01 | 0,59<<* | 0,64<<* | 0,92 | 1 | 0,53<<* | 0,65<<* | 1.05 |
| Exon 17 | 0,82 | 0,85 | 0,76 | 0,92 | 1 | 0.96 | 0.96 | 1.1 |
| Exon 1  AFF2 | 0 | 0,21 | 0,29 | 0 | 0.17 | 0,23>> | 0,23>> | 0 |
| Exon 1  AFF2 | 0 | 0 | 0,2>> | 0 | 0 | 0 | 0.2 | 0 |
| Exon 1  AFF2 | 0 | 0 | 0,13>> | 0 | 0 | 0 | 0.13 | 0 |
| Exon 1  AFF2 | 0 | 0 | 0 | 0 | 0 | 0 | 0 | 0 |
| Exon 1  AFF2 | 0 | 0 | 0 | 0 | 0 | 0 | 0 | 0 |
| Xp22 | 0,73 | 0,37 | 0,39 | 0,5 | 0.77 | 0,38<<* | 0,48<< | 0.86 |
| **Probes** | **FXS1 UT**  **1^st^ exp.** | **FXS1 T1**  **1^st^ exp.** | **FXS1 T3**  **1^st^ exp.** | **FXS1 T8**  **1^st^ exp.** | **FXS1 UT**  **2^nd^ exp.** | **FXS1 T1**  **2^nd^ exp.** | **FXS1 T3**  **2^nd^ exp.** | **FXS1 T8**  **2^nd^ exp.** |
|  | ratio | ratio | ratio | ratio | ratio | ratio | ratio | ratio |
| Prom | 1.43 | 0,53<<* | 0,68<<* | 0,86<<* | 0.97 | 0,62<<* | 0,76<< | 1,04>> |
| Prom | 1.2 | 0,38<<* | 0,42<<* | 0,74<<* | 0.97 | 0,61<* | 0,70<< | 1.04 |
| Exon 1 | 1.07 | 0,55<<* | 0,57<<* | 0,94<< | 0.91 | 0,63<<* | 0,72<< | 1,03>> |
| Intron 1 | 1.88 | 0,62<<* | 0,66<<* | 0,96<<* | 1.18 | 0,74<<* | 0,98<< | 1,5>>* |
| Intron 1 | 1.18 | 0,48<<* | 0,46<<* | 1.04 | 0.98 | 0,67<<* | 0,63<<* | 1.07 |
| Intron 1 | 0.81 | 0,33<<* | 0,26<<* | 0,39<<* | 0.66 | 0,57<< | 0.61 | 0.65 |
| Intron 1 | 1.08 | 0,45<<* | 0,45<<* | 0,93<< | 1.03 | 0,58<<* | 0,75<< | 1.01 |
| Exon 7 | 0.7 | 0,41<< | 0,42<< | 0.65 | 0.7 | 0,37<<* | 0,44<< | 0,58<< |
| Exon 17 | 0.89 | 0.8 | 0.88 | 0.96 | 1.04 | 0,82<< | 0.88 | 1.04 |
| Exon 1  AFF2 | 0.17 | 0 | 0.1 | 0 | 0 | 0 | 0.1 | 0 |
| Exon 1  AFF2 | 0 | 0 | 0 | 0 | 0 | 0 | 0 | 0 |
| Exon 1  AFF2 | 0 | 0 | 0 | 0 | 0 | 0 | 0 | 0 |
| Exon 1  AFF2 | 0 | 0 | 0 | 0 | 0 | 0 | 0 | 0 |
| Exon 1  AFF2 | 0 | 0 | 0 | 0 | 0 | 0 | 0 | 0 |
| Xp22 | 0.61 | 0<<* | 0,22<<* | 0.52 | 0.56 | 0 | 0,22<<* | 0.52 |
| **Probes** | **FXS2 UT**  **1^st^ exp.** | **FXS2 T1**  **1^st^ exp.** | **FXS2 T3**  **1^st^ exp.** | **FXS2 T8**  **1^st^ exp.** | **FXS2 UT**  **2^nd^ exp.** | **FXS2 T1**  **2^nd^ exp.** | **FXS2 T3**  **2^nd^ exp.** | **FXS2 T8**  **2^nd^ exp.** |
|  | ratio | ratio | ratio | ratio | ratio | ratio | ratio | ratio |
| Prom | 1.21 | 0,72<<* | 0,99<< | 1.23 | 1.22 | 0,61<<* | 0,85<<* | 1.24 |
| Prom | 1.04 | 0,68<<* | 0,72<<* | 0.97 | 1.17 | 0,64<<* | 0,73<<* | 1.23 |
| Exon 1 | 1.05 | 0,61<<* | 0,81<< | 0.92 | 1.07 | 0,61<<* | 0,66<<* | 1.05 |
| Intron 1 | 1.41 | 0,87<<* | 1,14<< | 1.33 | 1.32 | 0,84<<* | 1,01<<* | 1.34 |
| Intron 1 | 0.99 | 0,58<<* | 0,72<< | 0.98 | 1.17 | 0,59<<* | 0,64<<* | 1.24 |
| Intron 1 | 1.04 | 0,57<<* | 0,86<< | 1.02 | 1.15 | 0,59<<* | 0,69<<* | 1.16 |
| Intron 1 | 0.63 | 0.55 | 0.7 | 0,75>> | 1 | 0,58<<* | 0,5<<* | 0.85 |
| Exon 7 | 0.16 | 0 | 0.17 | 0 | 0.29 | 0.22 | 0.23 | 0.26 |
| Exon 17 | 0.98 | 0,8<< | 0.97 | 0,88<< | 0.93 | 0.96 | 0.93 | 1 |
| Exon 1  AFF2 | 0.55 | 0,33<< | 0,31<< | 0,39<< | 0.48 | 0.48 | 0.5 | 0,64>> |
| Exon 1  AFF2 | 0.43 | 0,18<< | 0,19<< | 0,29<< | 0.51 | 0.43 | 0.54 | 0.54 |
| Exon 1  AFF2 | 0.13 | 0 | 0 | 0 | 0.19 | 0.2 | 0.22 | 0.18 |
| Exon 1  AFF2 | 0.46 | 0,25<< | 0,24<< | 0,33<< | 0,3<< | 0.2 | 0,21<< | 0.34 |
| Exon 1  AFF2 | 0 | 0 | 0 | 0 | 0.05 | 0.07 | 0.05 | 0 |
| Xp22 | 0 | 0 | 0 | 0 | 0 | 0.14 | 0.1 | 0 |

Note that the asterisks indicate that the magnitude of the probe ratio exceed the set of arbitrary border values. The white box represents levels of methylation lower that the 30%, light grey represents those between 30-70%, while dark grey those higher than 70%.
