# Supplementary material for: Genome-wide methylation analysis demonstrates that 5-aza-2-deoxycytidine treatment does not cause random DNA demethylation in fragile X syndrome cells
Source: Epigenetics Chromatin. 2016 Mar 24;9:12. doi: 10.1186/s13072-016-0060-x (PMC4806452; doi:10.1186/s13072-016-0060-x)
Supplement: Supplementary file 3 — 10.1186/s13072-016-0060-x MS-MLPA analysis of the PWS/AS locus on chromosome 15 before and after 7-day treatment with 5-azadC (T1) of three different normal control female cell lines (WTA, B and C). Only methylation-sensitive probes are listed. [file 13072_2016_60_MOESM3_ESM.docx]

**Additional file: Table S2.** MS-MLPA analysis of the PWS/AS locus on chromosome 15 before and after 7-days treatment with 5-azadC (T1) of three different normal control female cell lines (WTA, B and C). Only methylation-sensitive probes are listed.

| **Probes** | **WTA UT**  ratio | **WTA T1**  ratio | **WT B UT**  ratio | **WT B T1**  ratio | **WTC UT**  ratio | **WTC T1**  ratio |
| --- | --- | --- | --- | --- | --- | --- |
| 15-021.5 NDN | 0,47 | 0,24 | 0,36 | 0,22<< | 0,49 | 0,28 |
| 15-022.9 SNRPN | 0,55 | 0,34<< | 0,6 | 0,46 | 0,57 | 0,44<< |
| 15-022.9 SNRPN | 0,57 | 0,29<< | 0,59 | 0,44 | 0,55 | 0,41 |
| 15-022.9 SNRPN | 0,62 | 0,31<< | 0,6 | 0,4 | 0,53 | 0,38 |
| 15-022.9 SNRPN | 0,42 | 0<< | 0,48 | 0,27 | 0,41 | 0,24 |
| 15-022.9 SNRPN | 0,61 | 0,23<<* | 0,81 | 0,42<< | 0,61 | 0,45 |
| 15-023.2 UBE3A Exon 1 | 0 | 0 | 0 | 0 | 0 | 0 |

Note that the asterisks indicate that the magnitude of the probe ratio exceed the set of arbitrary border values. The white box represents levels of methylation lower that the 30%, light grey box represents those between 30-70% and dark grey box represents those higher than 70%.
