# Supplementary material for: Genome-wide methylation analysis demonstrates that 5-aza-2-deoxycytidine treatment does not cause random DNA demethylation in fragile X syndrome cells
Source: Epigenetics Chromatin. 2016 Mar 24;9:12. doi: 10.1186/s13072-016-0060-x (PMC4806452; doi:10.1186/s13072-016-0060-x)
Supplement: Supplementary file 4 — 10.1186/s13072-016-0060-x MS-MLPA analysis of the BWS/SRS locus on chromosome 11 before and after 7-day treatment with 5-azadC (T1) of three different normal control female cell lines (WTA, B and C). Only methylation-sensitive probes are listed. [file 13072_2016_60_MOESM4_ESM.docx]

**Additional file: Table S3.** MS-MLPA analysis of the BWS/SRS locus on chromosome 11 before and after 7-days treatment with 5-azadC (T1) of three different normal control female cell lines (WTA, B and C). Only methylation-sensitive probes are listed.

| **Probes** | **WTA UT**  ratio | **WTA T1**  ratio | **WT B UT**  ratio | **WT B T1**  ratio | **WTC UT**  ratio | **WTC T1**  ratio |
| --- | --- | --- | --- | --- | --- | --- |
| 11-002.0 H19 | 0,3 | 0 | 0,25 | 0 | 0,29 | 0,22 |
| 11-002.0 H19 | 0,5 | 0,21<< | 0,5 | 0,34<< | 0,52 | 0,32<< |
| 11-002.0 H19 | 0,53 | 0,38<< | 0,6 | 0,42<< | 0,58 | 0,44<< |
| 11-002.0 H19 | 0,56 | 0,36<< | 0,62 | 0,45<< | 0,64 | 0,41<< |
| 11-002.1 IGF2 | 0 | 0 | 0 | 0 | 0 | 0 |
| 11-002.7 KCNQOT1 | 0,58 | 0,43<< | 0,57 | 0,43<< | 0,53 | 0,45 |
| 11-002.7 KCNQOT1 | 0,48 | 0,34<< | 0,53 | 0,44 | 0,54 | 0,4<< |
| 11-002.7 KCNQOT1 | 0,6 | 0,38<< | 0,5 | 0,42 | 0,51 | 0,47 |
| 11-002.7 KCNQOT1 | 0,51 | 0,31<< | 0,6 | 0,42 | 0,55 | 0,44 |
| 11-002.9 CDKN1c | 0,16 | 0,18 | 0,15 | 0,14 | 0,15 | 0,19 |

The white box represents levels of methylation lower that the 30%, light grey box represents those between 30-70%.
