# Supplementary material for: Genome-wide methylation analysis demonstrates that 5-aza-2-deoxycytidine treatment does not cause random DNA demethylation in fragile X syndrome cells
Source: Epigenetics Chromatin. 2016 Mar 24;9:12. doi: 10.1186/s13072-016-0060-x (PMC4806452; doi:10.1186/s13072-016-0060-x)
Supplement: Supplementary file 5 — 10.1186/s13072-016-0060-x Distribution curve of beta values for each of nine different samples. Two main peaks, for beta = 0 and 0.8 < beta < 1, are visible; beta = 0 means absence of DNA methylation and beta = 1 means completely methylated DNA. [file 13072_2016_60_MOESM5_ESM.docx]

**
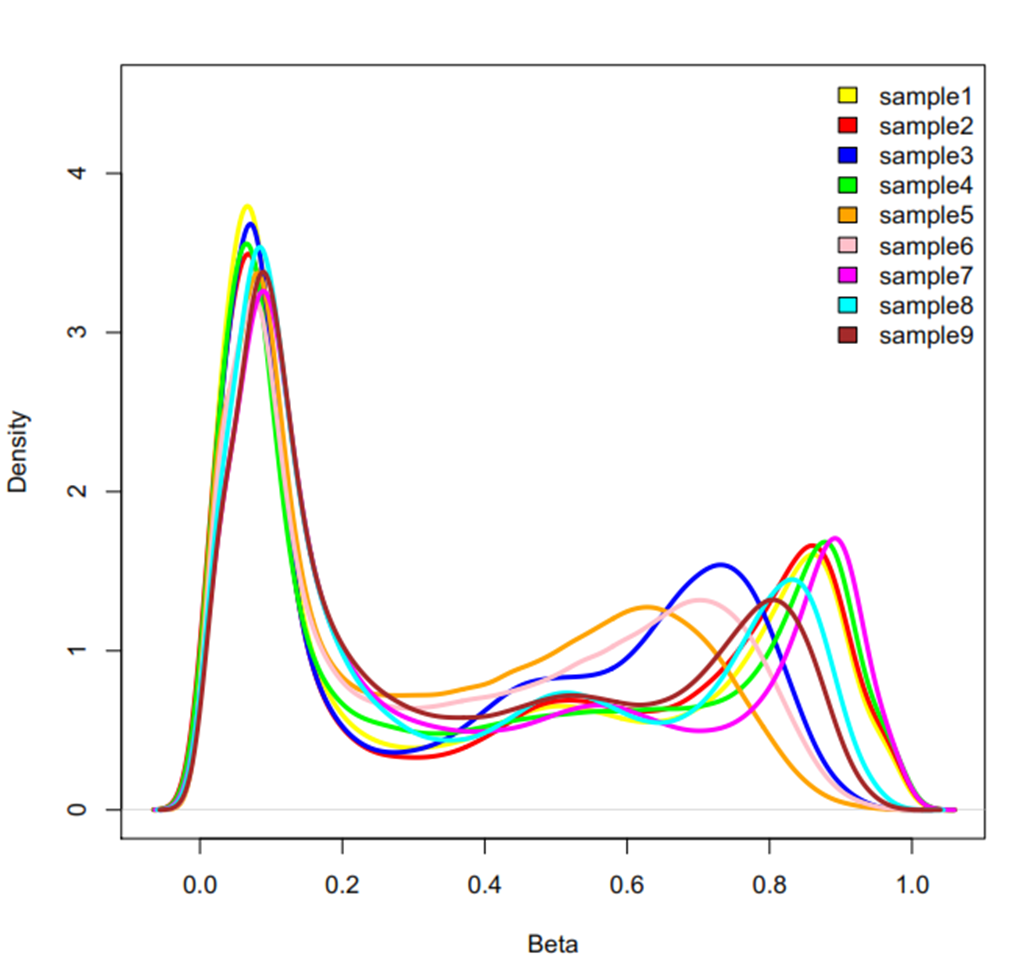
**

**Additional file: Figure S2.** *Distribution curve of beta-values for each of 9 different samples.* Two main peaks, for beta = 0 and 0.8 < beta < 1 are visible; beta=0 means absence of DNA methylation and beta=1 means completely methylated DNA.
