# Supplementary material for: Genome-wide methylation analysis demonstrates that 5-aza-2-deoxycytidine treatment does not cause random DNA demethylation in fragile X syndrome cells
Source: Epigenetics Chromatin. 2016 Mar 24;9:12. doi: 10.1186/s13072-016-0060-x (PMC4806452; doi:10.1186/s13072-016-0060-x)
Supplement: Supplementary file 6 — 10.1186/s13072-016-0060-x Heat map of the Pearson correlation analysis. Heat map illustrating the Pearson correlation coefficient between the nine different lymphoblastoid cell lines used for this study (columns from 1 to 9) and the data set of peripheral blood and iPS cells published by Alisch et al. [2013] (rows). A high correlation has been demonstrated with values ranged from 0.6 and 0.85 between our lymphoblastoid cell lines and those already published. Statistical significance was reached (p < 0.000001). [file 13072_2016_60_MOESM6_ESM.docx]

**
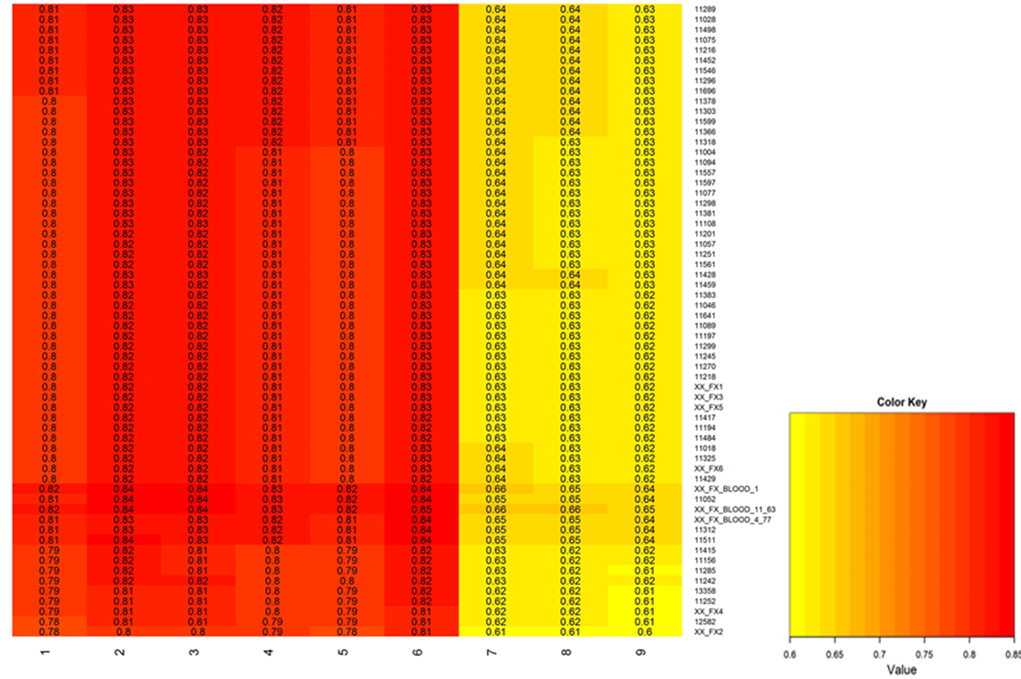
**

**Additional file: FigureS3.** *Heatmap of the Pearson correlation analysis.* Heatmap illustrating the Pearson correlation coefficient between the 9 different lymphoblastoid cell lines used for this study (columns from 1 to 9) and the data-set of peripheral blood and iPS cells published by Alisch et al. [2013] (rows). A high correlation has been demonstrated with values ranged from 0.6 and 0.85 between our lymphoblastoid cell lines and those already published. Statistical significance was reached (p < 0.000001).
